# A Comparison of Lower Dental Arch Changes Using Two Types of Space Regainers: A Removable Appliance with a Distalizing Screw and a Fixed Double-Banded Appliance

**DOI:** 10.1155/2022/4699516

**Published:** 2022-04-16

**Authors:** Shabnam Enteghad, Farzaneh Golfeshan, Ahmadreza Sardarian, Hooman Navaei

**Affiliations:** ^1^Orthodontics Research Center, School of Dentistry, Shiraz University of Medical Sciences, Shiraz, Iran; ^2^Dental Students' Research Committee, Department of Oral and Maxillofacial Surgery, School of Dentistry, Isfahan University of Medical Sciences, Isfahan, Iran

## Abstract

**Aim:**

The aim of this study was to compare lower dental arch changes using two types of space regainers, including a removable appliance with a distalizing screw and a fixed double-banded appliance.

**Methods and Materials:**

In this case-control study, the study sample was comprised of thirty-eight children with mixed dentitions, all of whom had unilateral space deficiency due to premature loss of the second deciduous molar in the mandibular arch. Patients were treated with either a removable appliance with a distalizing screw or a fixed double-banded space regainer (DBSR) (*n* = 19). Pre- and posttreatment dental casts and lateral cephalograms of patients were evaluated to compare the effects of the two space-regaining devices on the mandibular dental arch. The data were analyzed using paired and independent *t*-tests.

**Results:**

Available space, molar angle, IMPA, and the first molar distance to the mandibular plane and symphysis increased significantly in both groups (*P* < 0.001). The mean amount of IMPA changes was significantly greater in the distalizing screw group than in the DBSR group (*P* < 0.05). But, there were no statistically significant differences between the mean changes of available space, molar angle, and the first molar distance to the mandibular plane and symphysis in the distalizing screw and the DBSR group (*P* < 0.05). The DBSR group's treatment time was significantly shorter (*P* < 0.001).

**Conclusion:**

The removable device with a distalizing screw and the DBSR were both able to regain mild-to-moderate unilateral space loss, achieving an increase in molar angle, IMPA, and molar extrusion. However, treatment time with the DBSR was shorter and with less incisor tipping as a side effect.

## 1. Introduction

The proper management of space loss due to the premature loss of primary teeth is an important aspect of preventive and interceptive orthodontics. Dental caries is the most common cause of early loss of primary teeth. Other causes include trauma, ectopic eruption, congenital disorders, and early root resorption of primary teeth. Studies indicate that when a primary second rather than a primary first molar is lost, or if tooth loss happens at an earlier age, or in crowded dentitions, greater space loss is expected [1, 2].

The premature loss of primary teeth, particularly primary molars, allows mesial movement of the first permanent molar and distal drifting of the primary canine, which can disturb the integrity of the arch and may cause impaction or ectopic eruption of the second premolar [3, 4]. It has been seen that arch length decrease is greater in maxilla in comparison to the mandible, and distal drifting of the canine happens only in the lower arch [5]. Early loss of deciduous molars may also result in crowding, midline shift, and altering the development of normal occlusion [6]. There is an increased possibility of sagittal, vertical, as well as transversal malocclusion when premature loss of deciduous teeth happens [7]. It has been shown that early loss of deciduous molars can incite negative effect on oral health-related quality of life [8].

When space loss occurs, space regainers are required on a routine basis. Space regainers are the appliances that move the tooth mesially or distally to recover the lost space. These appliances help the permanent teeth erupt properly in their right place [2, 9]. If performed in carefully selected cases with correctly designed biomechanics, molar distalization can correct mild-to-moderate arch length discrepancies and also correct molar relations [10]. It is currently possible to evaluate the orthodontic treatment plan for the management of the lack of space in the arch with appropriate diagnostic and instrumental tests such as a single 3D survey to execute the complete treatment plan and a final rendering [11].

Different removable and fixed appliances are used for space regaining. Fixed space regainers are sliding loop regainer, open coiled space regainer, Gerber space regainer, double-banded space regainer, pendulum appliance, distal jet appliance, and lip bumper [2, 12]. Removable space regainers are as follows: C-space regainer, Hawley appliance with helical spring, fixed-removable Hawley appliance, lower Hawley appliance with split-acrylic spring, and lower Hawley appliance with sling-shot elastic [2, 12].

Removable devices may be used in the mandibular arch for moderate quantities of space recovery just as they are used in the maxillary arch, but they are generally less satisfying because, compared to the removable plate in the maxillary arch, they are more fragile and vulnerable to breakage. Lower removable plates do not have palatal anchorage support and may not fit as well [13], and it is hard to control their displacement. Also, due to the tenderness of the gingival tissues and the undercuts on the lingual side of the mandible, the acrylic component must be small in the lower arch. Consequently, there is limited space available for spring construction. These aspects explain why there is better control over individual teeth and more can be accomplished in the maxilla than in the mandible with removable plates. In addition, the reason that so many lower plates are irregularly worn and patient cooperation is low can become clear [14].

Therefore, numerous intra-arch devices have been implemented that have reduced dependency on patient cooperation. One of these devices is the double-banded space regainer, which is composed of two bands and a nickel titanium (NiTi) spring, which can recover space due to the superelasticity of the NiTi wire. For the first time in 2012, Chalakkal et al. [15] reported the use of “Double-banded space regainer” in a case where early exfoliation of the primary maxillary molar had resulted in mesial migration of the permanent first molar and space deficiency for premolar eruption. Later in 2016, Patil et al. [16] described a case of space regaining, achieved with the distal movement of two teeth using the double-banded space regainer with some modifications.

The removable plate with a distalizing screw has been shown by da Costa et al. [17] to recover space loss due to early loss of primary molars in the lower arch. They did, however, report issues with the appliance, such as soft tissue irritations and lesions. On the other hand, just a few case reports [15, 16] about the double-banded space regainer have been published, and this device has only been employed in the upper arch. Due to the lower need for patient cooperation and activations in the double-banded device than in the removable plate, and also the problems associated with the removable plate in the lower arch, we sought to compare these two types of devices in the form of a case-control study. The present study aimed to compare lower dental arch changes using two types of space regainers, including a removable appliance with a distalizing screw and a fixed double-banded appliance, to select the best device in terms of efficiency in the clinic.

## 2. Materials and Methods

The protocol for this study was approved by the Shiraz University of medical science ethics committee with number IR.SUMS.DENTAL.REC.1398.111.

The study sample was comprised of two groups of 19 children between the ages of 7 and 11. One group was treated with a removable appliance with a distalizing screw and the other with a fixed double-banded space regainer for molar distalization. The subjects were selected retrospectively, and the treatments were provided by one clinician. All subjects met the following criteria:

1. The children were 7 to 11 years of age, all of whom had mixed dentitions.
2. All of the children had 3–5 mm reduced mandibular perimeter due to premature loss of second primary molar.
3. In all subjects, the mandibular permanent incisors and first molars had erupted, and primary or permanent canine had erupted on the space loss side.
4. The space loss was unilateral in all cases.
5. Patients had normal skeletal growth patterns.
6. Second permanent molar was in a position apical to the first molar's cementoenamel junction (CEJ).
7. No history of orthodontic treatment before.
8. Good oral hygiene and cooperation.
9. The mesial angulation of none of the first permanent molars differed by more than 10 degrees from normal.

The above inclusion criteria were confirmed according to the information in the patients' records and the pretreatment documents, including intraoral photographs, orthopantomograms, lateral cephalograms, dental models, and the results of space analysis. However, the measurements for space analysis were done again on pretreatment dental models, and the results of space analysis in patients' records were confirmed.

Sample size calculation was based on the study of da Costa et al. [17]. With an alpha level of 0.05 and a statistical power of 90%, a minimum of 19 subjects in each group was required to detect a minimum difference of 1.5 mm between groups for the amount of regained space as the primary outcome variable, with a standard deviation (SD) of 1.39 mm.

### 2.1. The Design of the Removable Appliance with a Distalizing Screw

**Figure 1:**
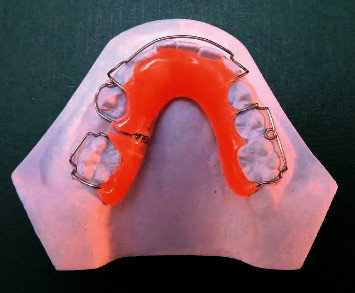
Removable plate with a distalizing screw.

The device was fabricated on the working cast and featured a Hawley's loop (labial bow), three or four retention clasps, one distalizing screw on the side of space loss, and an acrylic lingual baseplate (Figure 1). Information was given to patients and their parents about the appliance usage, its placement and removal, and how to maintain oral hygiene. Patients were advised to wear the space regainer full-time, except when eating, brushing their teeth, and cleaning the appliance, in order to maximize the device's effectiveness. For a week, the device stayed passive in the oral cavity so that the children could overcome the initial difficulties. After that, patients and parents were instructed to activate the distalizing screw a quarter of a turn (0.25 mm) two times a week. Patients were recalled every month, and the retention clasps were adjusted at each appointment until the required space for the eruption of the second premolar was regained.

### 2.2. The Design of the Double-Banded Space Regainer

**Figure 2:**
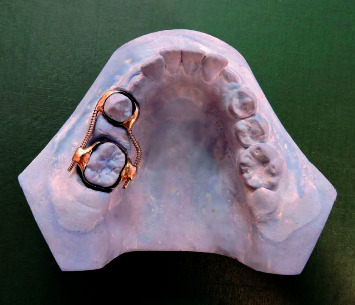
The double-banded space regainer.

The design of this appliance was similar to that described by Chalakkal et al. [15], with the exception of some modifications. The permanent first molar was banded (American Orthodontics Inc.) with buccal and palatal welded molar tubes (1.1 mm in diameter, 4.2 mm in length). The first premolar, or primary first molar, was also banded. An alginate impression was taken. Impression, along with bands, was washed and disinfected with sodium hypochlorite solution. The model was made with dental stone, keeping the bands in the impression. On the working model, two stainless steel (0.9 mm) wires were adapted and soldered to the bands of the first premolar or primary molar buccally and palatally, extending posteriorly to insert into the molar tubes of the permanent first molar. The Ni-Ti open coil springs (G&H® Inc., USA; 0.010 in diameter; 0.045 in lumen) were cut 4 mm longer than the distance between the anterior stops, which were the solder joints, and the molar tubes were cut posteriorly and incorporated into the stainless steel wires. The device (DBSR) was cemented to the teeth using glass ionomer cement while the springs were held in compression (Figure 2). Patients were recalled every month, and the springs were reactivated if needed, until the required space for the eruption of the second premolar was regained. This was assessed clinically. At this time, posttreatment dental casts and radiographs were provided. After that, the devices remained passive as space maintainers until the eruption of the second premolar happened.

### 2.3. Cast Analysis

**Figure 3:**
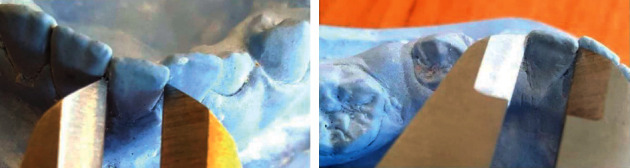
Measuring the mesiodistal width of the central (left) and lateral (right) incisor with digital calipers.

**Figure 4:**
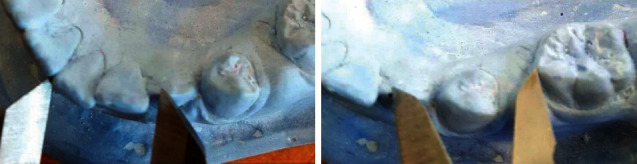
Determining the laterally adjusted point to the distal surface of the lateral incisor with calipers (left). Measuring the distance between the laterally adjusted point to the distal surface of the lateral incisor to the mesial surface of the first permanent molar to determine the available space (right).

Alginate impressions (Tropicalgin; Zhermack SpA, Badia Polesine, Italy) were taken before placement of space regainers (*T*_1_) and at the end of active space-regaining treatment (*T*_2_), and stone models were made using type IV dental gypsum (GC Corp., Tokyo, Japan). To determine the required space for the eruption of permanent canine, first and second premolar, the modified Tanaka-Johnston equation was used [18, 19]. The available space for the eruption of these teeth in the hemiarch of space loss was determined on the stone models. For this purpose, the distance between the distal surface of the lateral incisor and the mesial surface of the first permanent molar was measured using a digital caliper. In the case of incisor crowding, to measure the available space, a laterally adjusted point to the distal surface of lateral incisor was determined. To determine this point, the calipers was applied on the midline with the measurement of the sum of the mesiodistal widths of central and lateral incisors in the hemiarch of the space loss (Figures 3 and 4) [17]. Using this method, the available space was measured on pre- and posttreatment stone models, and the difference determined the recovered space.

### 2.4. Cephalometric Analysis

All lateral cephalograms were acquired using one imaging system (Planmeca X Proline cephalostat, Instrumentarium corp. Imaging Division, Tuusula, Finland) at 80–85 kV with a source-midsagittal plane distance of 1.5 m and a film-midsagittal plane distance of 15 cm so that the radiographic magnification factor was similar in all cephalograms.

**Figure 5:**
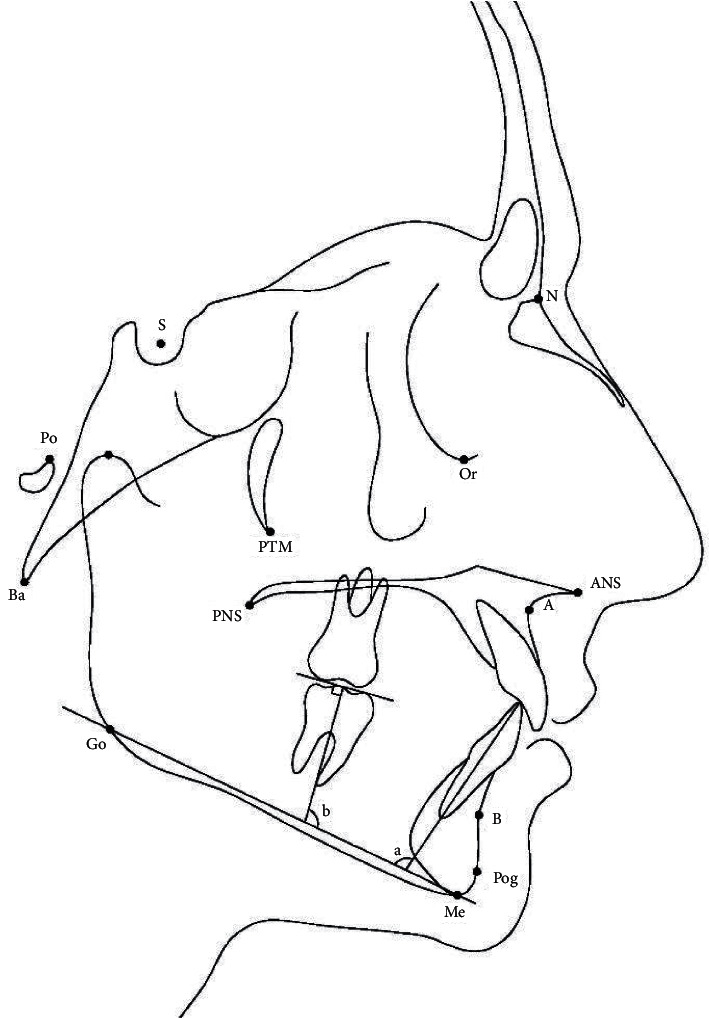
(a). IMPA. (b). Molar angle.

**Figure 6:**
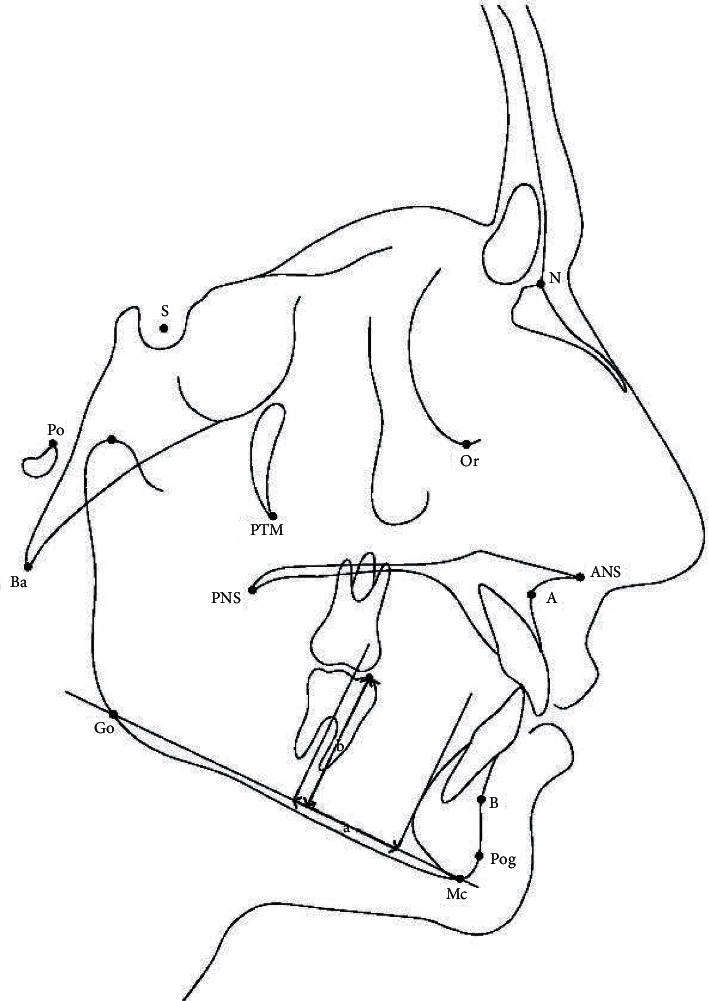
(a). Molar distance to symphysis. (b). Distance between the mesiobuccal cusp tip of the first permanent molar and the mandibular plane.

Pre- (*T*_1_) and posttreatment (*T*_2_) cephalograms were used for cephalometric measurements. The lateral cephalograms were traced on acetate paper sheets using a lead pencil. The lower incisor angulation was measured as the angle formed by the long axis of the incisor to the mandibular plane (IMPA). The difference between the IMPA measurements on pre- and posttreatment cephalograms indicated the proclination of the lower permanent incisors that worked as part of the anchorage for the space-regaining device (Figure 5). To determine the molar angle, first a tangent was formed to the occlusal surface of the first permanent molar, then a perpendicular line passing through the furcation was drawn. The superior-anterior angle was formed by this line, and the mandibular plane was measured to determine the molar angle (Figure 5) [20]. Molar angle changes demonstrated the first molar's inclination. Molar bodily movement was measured as the distance from two reference planes: one was a tangent to the symphysis posterior margin that was perpendicular to the mandibular plane, and the other was a parallel line that passed through the furcation of the first permanent molar. The changes in the distance between these two reference points before and after treatment showed the amount of bodily movement of the first molar by the space-regaining appliance (Figure 6) [20]. The vertical position of the first permanent molar was determined by measuring the distance between the occlusal surface of the mesiobuccal cusp of the first permanent molar and the mandibular plane (Figure 6). Molar vertical changes determined the extrusion effect of the appliance. The measurements shown in Table 1 were used to analyze the changes in the dental arches in the pre- and posttreatment phases.

The duration of treatment was determined in months for each patient. This was the period of time between the first activation of each device and when the required space for the eruption of the second premolar was regained, and the treatment was completed.

### 2.5. Statistical Analysis and Error of the Method

The data were analyzed using SPSS version 26. The mean and standard deviation (SD) were calculated for each variable used in analyzing the casts and lateral cephalograms. The distributions of all variables were normally based on the Shapiro-Wilk test. Paired *t*-test was used to compare pre- to posttreatment variables, and independent *t*-test was used to assess whether differences in means between the groups achieved statistical significance. The level of significance was set at 0.05.

In order to determine the error of the method, two weeks after the first measurements, records of 10 randomly selected subjects were remeasured by the same examiner. The intraexaminer error was assessed using the intraclass correlation coefficient (ICC). The ICC was in the range of 0.92–0.98. Hence, the measurements enjoyed sufficient reliability.

## 3. Results

The study sample consisted of 19 subjects (11 girls and 8 boys) with a mean age of 10.0 (SD 1.38) in the distalizing screw and 19 subjects (10 girls and 9 boys) with a mean age of 9.9 (SD 0.84) years in the DBSR group. The two groups were homogeneous because no significant difference was found for the variables measured at *T*_1_ (pretreatment) between the two groups (*P* > 0.05).

Dental cast measurements showed that the available space increased significantly in both groups (*P* < 0.001). There was no significant difference in the amount of space regained between the distalizing screw (3.53 mm) and the DBSR (3.51 mm) groups (*P*=0.974).

Cephalometric measurements showed that molar angle increased significantly in both groups (*P* < 0.001) which means that both devices tipped the first molar distally. The DBSR group had a greater mean change in molar angle (3.1°) than the distalizing screw group (2.2°). However, the difference between groups was not statistically significant (*P*=0.078).

Results showed that the incisor mandibular plane angle, or IMPA, increased in both groups by the space-regaining treatment (*P* < 0.001). The mean amount of IMPA change was significantly greater in the distalizing screw (3.3°) group than in the DBSR (2.1°) group (*P* < 0.05).

The amount of molar distance from the posterior margin of the symphysis increased significantly in both groups (*P* < 0.001). There was no statistically significant difference in the mean distance changes between the distalizing screw and the DBSR group (*P*=0.5).

There was a significant increase in the distance between the mesiobuccal cusp tip of the first molar and the mandibular plane in both the distalizing screw and DBSR groups (*P* < 0.001). The average increase was 2.0 mm in the distalizing screw and 2.3 mm in the DBSR group, with no significant difference between the groups (*P*=0.317).

Means, standard deviations, and comparison of the pre- and posttreatment measurements are shown in Table 2. Changes in cast and cephalometric variables for the two treatment groups are compared in Table 3.

The average molar distalization time for the distalizing screw group was 11.6 ± 1.96 months, whereas in the DBSR group, the corresponding time was 8.1 ± 2.21 months. Thus, the treatment time for the distal molar movement was significantly shorter for the DBSR than for the distalizing screw group (*P* < 0.001).

## 4. Discussion

The present study evaluated the effects of two types of space-regaining devices on the mandibular dental arch.

Early loss of primary teeth can cause a variety of problems, including space loss, one of the most important [22]. Space regaining, as the main treatment for this problem, can be done in both upper and lower arches. However, space regaining in the lower arch is more difficult than in the upper arch [10]. The mandibular molar is known to be the hardest tooth to move, which is due to the broad root area and root morphology, as well as the higher density of bone in the lower jaw compared with the upper jaw [23, 24]. Lip bumpers, lingual arches, and removable devices with screws or springs are the most widely used intraoral devices for space regaining in the mandible [10].

A removable appliance with a jackscrew is the most commonly used appliance for space regaining in the clinic, which relies on patient cooperation for its effectiveness. In the mandible, it is known that removable appliances with active springs or screws can cause patient compliance problems due to lack of adequate retention and easy dislodgement as a result of tongue movement and also irritation of the lingual gingival tissues [14]. On the other hand, these appliances have some advantages over fixed devices. One of the most important is that the appliance and the teeth could be better cleaned. Patients treated with removable appliances display better oral hygiene, less plaque, and less gingival inflammation [25] while, in patients with fixed orthodontic appliances, oral hygiene becomes more difficult and a prevalent finding is the decalcification of the enamel surface (manifested as a white spot lesion) adjacent to these appliances [26]. Studies have shown that salivary bacterial and candida colonization causing vulnerability to developing caries and candida infection is higher in patients with fixed rather than removable appliances [27, 28].

Since the use of removable appliances requires considerable patient cooperation, in the present study, we compared a simpler noncompliance appliance (the double-banded space regainer) with the conventionally used removable plate. The DBSR uses Ni-Ti coil springs for molar distalization, which can generate continuous light forces over a wide range of activation [29]. In addition, this appliance does not need as many activations as the distalizing screw does. However, in this study, one or more spring reactivations were necessary in some patients as the magnitude of forces applied to the teeth decreases when the teeth move and springs decompress [30].

In the present study, to determine the required space, the modified Tanaka-Johnston equation was used, which has been shown to accurately estimate the size of unerupted canines and premolars in the Iranian population [19]. The results indicated no statistically significant difference between the mean recovered spaces of the distalizing screw and the double-banded space regainer. The mean amount of space regained in both groups was 3.5 mm which means that both devices seem to be effective in regaining mild-to-moderate unilateral space loss.

The amount of molar distance from the posterior margin of the symphysis increased significantly in both groups, and there was no statistically significant difference between the two study groups. These results mean that a noticeable amount of space recovered in both groups was due to the distal bodily movement of the first permanent molar (2.7 and 2.4 mm in the distalizing screw and the DBSR groups, respectively). It should be noted that in the analysis of lateral cephalometry images, evaluating bilateral structures such as first molars can be challenging due to the presence of two shadows of these structures. In the current study, the selection of subjects with unilateral space loss made it possible to distinguish between the right and left first molars more easily. This means that the mesially located first molar in the lateral cephalogram was considered as the molar on the space loss side. Moreover, unlike the molar on the space loss side, the position of the molar on the opposite side did not change considerably between the pre- and posttreatment cephalograms. Another helpful criterion for differentiating the two molars was that the image of the left molar was usually less distorted as it was closer to the film.

Intraoral molar distalization has some side effects, including tipping of incisors or premolars (or both) in different amounts depending on the choice of distalization appliance [31]. In the present study, the mandibular incisors in both study groups experienced an increase in proclination. Forward movement of mandibular incisors has been seen in studies of molar distalization with lip bumper [32], nickel titanium open-coil springs [33, 34], and removable plates with distalizing screws [17].

While there was a significant increase in IMPA after treatment with DBSR in the current study, there are no previous data in the literature about changes in IMPA after treatment with this appliance. Looking at the present results, by using DBSR, molar distalization seems to have occurred with less proclination of the lower anteriors with respect to the distalizing screw. It can be due to the fact that in the distalizing screw, the acrylic plate is in direct contact with the lingual surfaces of incisors and transmits the force generated by the Jack screw to the teeth in a labial direction. It can also be related to the probable misfit and displacement of the acrylic plate. When using mandibular removable plates in the clinic, it is often observed that after some time, the acrylic plate may not fit well; this is perhaps because the molar does not distalize as much as the Jack screw is opened, which leads to a misfit of the plate. In this situation, the anterior part of the acrylic plate contacts the incisors closer to the incisal edge and further from the center of rotation of the tooth, causing more tipping of these teeth. Another reason that may explain this result is the fact that when lighter forces are applied, anchorage loss is expected to be less [35]. The Ni-Ti coil springs used in the DBSR produce light continuous forces which are optimal for orthodontic tooth movement and probably cause less anchorage loss. Thus, the double-banded space regainer might be favorable in cases of mandibular incisor protrusion.

Molar tipping is another common finding in cases of molar distalization. In the present study, the removable plate with a distalizing screw and the double-banded space regainer both tipped the first permanent molar distally and increased the molar angle. In the study of da Costa et al. [17], distal tipping of mandibular molars by the distalizing screw was reported as well. However, in the studies of Chalakkal [15] and Patil [16] on the DBSR, no observable tipping on the permanent first molar was seen. The difference between these two studies and the present study can be related to the fact that in their studies, the DBSR was used in the maxillary dental arch, which has lower bone density than the mandible [36]. The compact bone and oblique ridges in the mandible make bodily movement of molar roots difficult, leading to tipping of the tooth [37]. In our study, the amount of first molar tipping was greater in the DBSR group (3.1°) than in the distalizing screw group (2.2°). However, the difference that existed between the two groups was not statistically significant.

Considering the vertical changes, both appliances extruded the mandibular molar and no significant difference was observed between the amounts of extrusion in the two study groups. Extrusion of the first molar was also observed in the study of Byloff et al. [33] with the Franzulum appliance, which uses nickel titanium coil springs for mandibular molar distalization. The side effect of tooth extrusion occurs with all simple tip-back-uprighting springs when correcting the axial inclination of the molar. Therefore, controlling vertical movements (extrusion) of molars should be considered in molar uprighting. Extrusion of mandibular molar may be acceptable when the tipped molar is below the functional occlusal plane in some early orthodontic treatments [38] and desirable in patients with deep bite.

The basis of an efficient orthodontic treatment is a good understanding of the tooth movement rate and the amount of anchorage loss as well [35]. In the present study, statistically significant differences between the distalizing screw and DBSR groups were observed when comparing the period of use of the space-regaining device. The DBSR was used for a shorter time than the distalizing screw. This can be related to the fact that the DBSR appliance cannot be removed that ensures continuous forces applied to the target teeth with no reliance on special patient compliance. However, in comparison to other studies using the DBSR for maxillary molar distalization [15, 16], space recovery with the DBSR took more time in our study. This may be due to the increased trabecular structure and resilient spongy bone in maxilla [37]. The cortical bone of the mandible is denser than that of the maxilla [24], and the remodeling rate is slower in the mandible [39]. Therefore, it has been observed that teeth move faster and further in the upper arch than in the lower arch [37]. Another factor is the patients' age, which was lower in those studies. The advantage of earlier molar distalization is that the permanent first molar roots are incomplete, and it is easier to tip or move the tooth bodily [16].

### 4.1. Limitations

This was a retrospective study and also had the limitation of sample size, which should be overcome by future prospective studies with larger sample sizes. Also, it is suggested to evaluate movements of the tooth mesial to the space loss region in future studies.

## 5. Conclusion

Within the limitations of this study, it was concluded that the removable device with a distalizing screw and the DBSR are both able to regain mild-to-moderate unilateral space loss when used in patients with mixed dentition, resulting in an increase in molar angle, IMPA, and molar extrusion. But, the DBSR seemed to be more efficient since it had a shorter treatment time and a lower side effect of incisor tipping. However, the removable device can be implemented in patients with low incisor protrusion and good cooperation, with the advantage of better oral hygiene maintenance.

## Figures and Tables

**Table 1:** Measured variables and their definitions.

Variable	Definition
Available space (mm)	Space measured between the distal surface of the lateral incisor and the mesial surface of the permanent first molar
Required space (mm)	Sum of mesiodistal width of permanent canine and premolars estimated by Tanaka-Johnston method
Molar angle (degree)	Angle formed by the long axis of the mandibular first permanent molar and mandibular plane
IMPA (degree)	Angle formed by the long axis of mandibular incisors and the mandibular plane
Molar to symphysis (mm)	Distance between mandibular first permanent molar and posterior border of symphysis
Molar to mandibular plane (mm)	Distance between mesiobuccal cusp tip of mandibular first permanent molar and the mandibular plane

The definitions given in this table are derived from Werner et al. [21] and da Costa et al. [17].

**Table 2:** Comparison of the pre- and posttreatment mean values in each group.

Variable	Distalizing screw (*n* = 19)			DBSR (*n* = 19)			Homogeneity
	Pretreatment	Posttreatment	*P* value	Pretreatment	Posttreatment	*P* value	
	Mean ± SD	Mean ± SD		Mean ± SD	Mean ± SD		
Required space (mm)	24.53 ± 1.87			23.32 ± 1.65			0.43
Available space (mm)	21.40 ± 2.64	24.93 ± 2.75	<0.001^*∗*^	20.17 ± 2.31	23.69 ± 2.32	<0.001^*∗*^	0.52
Molar angle (degree)	88.07 ± 8.83	90.30 ± 8.91	<0.001^*∗*^	91.68 ± 9.31	94.80 ± 9.22	<0.001^*∗*^	0.43
IMPA (degree)	90.60 ± 6.87	93.93 ± 7.26	<0.001^*∗*^	88.00 ± 7.21	90.14 ± 7.27	<0.001^*∗*^	0.29
Molar to symphysis (mm)	20.00 ± 4.95	22.70 ± 4.58	<0.001^*∗*^	22.36 ± 2.73	24.76 ± 2.71	<0.001^*∗*^	0.33
Molar to MP (mm)	28.07 ± 3.97	30.07 ± 4.39	<0.001^*∗*^	27.71 ± 3.67	30.06 ± 3.68	<0.001^*∗*^	0.26

DBSR: double-banded space regainer; SD: standard deviation; IMPA: incisor mandibular plane angle; MP: mandibular plane; ^*∗*^*P* < 0.05.

**Table 3:** Comparison of cast and cephalometric changes (*T*_2_ − *T*_1_) between two groups.

Variable	Distalizing screw (*n* = 19)	DBSR (*n* = 19)	95% CI		*P* value
	*T*_2_ − *T*_1_ mean ± SD	*T*_2_ − *T*_1_ mean ± SD	Lower	Upper	
Available space (mm)	3.53 ± 1.16	3.51 ± 0.82	−0.75	0.87	0.974
Molar angle (degree)	2.23 ± 1.40	3.12 ± 1.19	−1.88	0.11	0.078
IMPA (degree)	3.33 ± 1.71	2.14 ± 1.05	0.10	2.28	0.033^*∗*^
Molar to symphysis (mm)	2.70 ± 1.45	2.41 ± 0.71	−0.58	1.17	0.5
Molar to MP (mm)	2.00 ± 0.73	2.35 ± 1.09	−1.05	0.35	0.317

DBSR: double-banded space regainer; CI: confidence interval; SD: standard deviation; IMPA: incisor mandibular plane angle; MP: mandibular plane; *T*_1_: pretreatment; *T*_2_: posttreatment; ^*∗*^*P* < 0.05.

## Data Availability

Data are available from the corresponding author upon request.
